# Imaging through scattering medium by adaptive non-linear digital processing

**DOI:** 10.1038/s41598-018-28523-6

**Published:** 2018-07-12

**Authors:** Saswata Mukherjee, Joseph Rosen

**Affiliations:** Department of Electrical and Computer Engineering, Ben-Gurion University of the Negev, P.O. Box 653, Beer-Sheva, 8410501 Israel

## Abstract

Scattering media have always posed obstacles for imaging through them. In this study, we propose a single exposure, spatially incoherent and interferenceless method capable of imaging multi-plane objects through scattering media using only a single lens and a digital camera. A point object and a resolution chart are precisely placed at the same axial location, and light scattered from them is focused onto an image sensor using a spherical lens. For both cases, intensity patterns are recorded under identical conditions using only a single camera shot. The final image is obtained by an adaptive non-linear cross-correlation between the response functions of the point object and of the resolution chart. The clear and sharp reconstructed image demonstrates the validity of the method.

## Introduction

Imaging through a scattering medium in the visible region of the electromagnetic spectrum is a challenging task. Researchers started working on understanding and finding solutions to the problem in the mid 60’s^1–3^. Methods of imaging through scattering media have a wide range of applications in the fields of biomedical imaging^4^, imaging through fog^5^, astronomical imaging and imaging through turbid media^6^. Different coherent digital holography techniques for imaging through scattering media^2–8^ have been demonstrated. While scatterers are mostly considered to be a nuisance for imaging, they have also been used as a tool for imaging in some cases, such as the improvement of lateral resolution^9,10^. Scattering masks with controllable scattering ranks have been used to improve axial^11^ and lateral^12^ image resolutions.

Lasers have a relatively high optical power output, and thus are the primary choice for the light source in systems that image through diffusive media^13^. However, the laser’s higher optical power comes with the disadvantages of higher cost and undesirable imaging effects, such as speckles, ringing artifacts and a narrow bandwidth. A simple lensless incoherent setup capable of imaging objects through scatterers^14^ using a Fienup type algorithm^15^ for reconstructing the image has been proposed and demonstrated. Another incoherent microscopy^16^ technique capable of achieving super-resolution with scattering masks, which uses the Richardson–Lucy deconvolution^17^ algorithm for reconstruction, has been demonstrated. Compressive sensing has also played a crucial role in 3D imaging through scattering media using incoherent sources, as presented by *Antipa et al*.^18^.

Wavefront shaping^19^ using real-time dynamic feedback^20,21^ can also pave the way for imaging through diffusers. Several other methods, including Monte-Carlo analysis in two-photon fluorescence^22^, confocal imaging using an annular pupil^23^, absorption studies^24^ and transmission matrix analysis^25^ have also been proven to achieve similar goals. A coherent digital holographic technique^26^ capable of 3D imaging and phase retrieval through scatterers has also been demonstrated. Another coherent digital holography technique has been demonstrated for 3D imaging and phase retrieval, but its optical configuration is complex with many optical components^27^. An adaptive optics based incoherent digital holography technique^28,29^ using the principles of Fresnel incoherent correlation holography^30^ also has the ability to image through turbid media, using a guide-star.

Another lensless incoherent 3D imaging^31^ technique for retrieving objects embedded between dynamic scatterers has been proposed and experimentally demonstrated, but it requires an off axis reference point for calibration purposes, thus making the setup difficult to align. However, the use of a reference point together with an object leads to certain limitations on the setup, such as a limited field of view. Recently, a scatter-plate microscope^32^, which uses the scatterer as a tunable objective lens of a microscope and can detect objects through scattering layers, has been demonstrated, but the reconstruction procedure has not shown 3D imaging capability. Similarly, another incoherent imaging technique^33^ which utilizes a known reference object to reconstruct the object of interest was recently demonstrated, but 3D imaging was not shown. A phase-diversity non-invasive speckle imaging method^34^ which can image through a thin scatterer was also reported. However, the method requires multiple camera shots from different positions of the sensor and 3D imaging is not demonstrated. A high-speed, full-color image technique^35^ through a standard scattering medium using broadband white-light as the illumination source was shown recently; this technique can reconstruct objects hidden behind turbid media using a reconstruction algorithm. More recently it was shown that a broadband image of an object can be reconstructed from its speckle pattern, where the scattering medium plays the role of an imaging lens^36^.

In this study, we present a new method of imaging through a scattering medium. The method is based on characterization of the scatterer with a guide-star. However, the linear cross-correlation with the response to the guide-star^37^ is replaced by an adaptive non-linear reconstruction process. Thus, instead of using two camera shots with two independent scatterers^37^, the non-linear digital process makes it possible to reconstruct the hidden multi-plane object from a single camera shot and without using an interferometer. Unlike our previous work^37^, the optimal parameters of the non-linear reconstructing process are chosen by optimizing a blind figure-of-merit, without the need for any prior knowledge about the covered object. Although characterizing the scatterer with a guide-star limits the use of the method to certain applications, the use of a guide-star makes it possible to do 3D imaging^37^.

## Methodology

**Figure 1:**
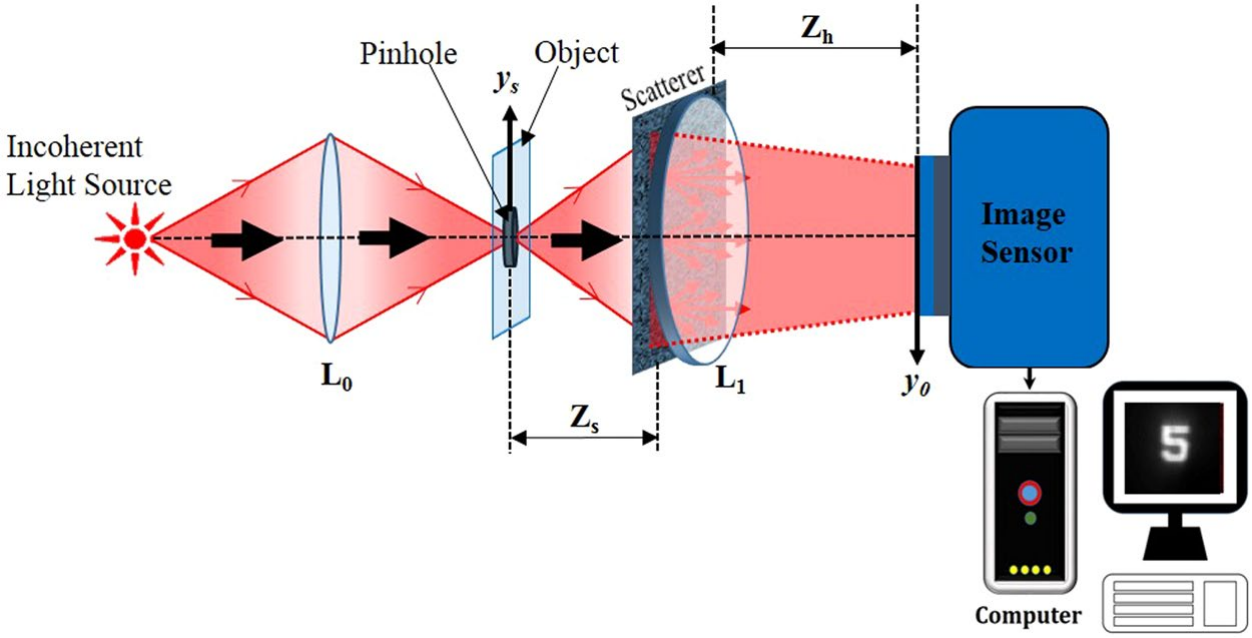
Optical configuration of the imaging system with the scatterer.

The optical setup for the proposed technique is shown in Fig. 1. The light diffracted by the point object is modulated by a scatterer located at a distance *z*_*s*_ from the point object. A refractive lens L_1_, placed at close proximity to the scatterer is used to focus the modulated light onto the image sensor. Lens L_1_ has a focal length *f* = (1/*z*_*s*_ + 1/*z*_*h*_)^−1^, where *z*_*h*_ is the separation between the lens L_1_ and the image sensor. Without the scatterer, a focused image of the point object is obtained on the image sensor. It is well-known^38^ that when a positive lens is illuminated by a quasi-monochromatic point source, a 2D Fourier transform of a transparency (multiplied by some quadratic phase function) is obtained on the image plane of the source, when the transparency is placed anywhere between the source and the image point. The center of the Fourier transform corresponds to the image of the point source. Hence, if the source is at the point ${\bar{r}}_{s}=({x}_{s},{y}_{s})$, the intensity at the sensor plane will be located at ${\bar{r}}_{o}={\bar{r}}_{s}{z}_{h}/{z}_{s}$. The intensity at the sensor plane, known as the point spread function^37^ (PSF) is given by,

1 $$\begin{array}{ccc}{I}_{PSF}({\bar{r}}_{0}) & = & C{|\nu [\frac{1}{\lambda {z}_{h}}]\overline{)\Im }[L(\frac{{\bar{r}}_{s}}{{z}_{s}})\exp (i{{\rm{\Phi }}}_{r})]|}^{2}\\  & = & C{|\nu [\frac{1}{\lambda {z}_{h}}]\overline{)\Im }[\exp (i{{\rm{\Phi }}}_{r})]|}^{2}\ast \delta ({\bar{r}}_{0}-\frac{{z}_{h}}{{z}_{s}}{\bar{r}}_{s}),\end{array}$$

where *C* is a constant, *ν* is a scaling operator performing the operation *ν*[*a*]*f(x)* = *f(ax)*, $\overline{)\Im }$ indicates a 2D Fourier transform performed on the caustic phase profile, ${{\rm{\Phi }}}_{r}$ of the scatterer. The star represents 2D convolution and *L*(∙) is a linear phase function such that $L(\bar{s}/z)=\exp [i2\pi {(\lambda z)}^{-1}({s}_{x}x+{s}_{y}y)]$.

A 2-D object can be represented by a set of independent points, and is mathematically expressed as,

2 $$o({\bar{r}}_{s})=\sum _{j}^{N}{c}_{j}\delta ({\bar{r}}_{s}-{\bar{r}}_{j}),$$

where each *c*_*j*_ is a positive real constant. The object is placed at the same axial location as the point object. The light emitted from the object passes through the same scattering sheet before reaching the image sensor. Since the optical system is linear and space-invariant, the intensity profile captured at the image sensor is given by,

3 $${I}_{Obj}({\bar{r}}_{0})=\sum _{j}{c}_{j}{I}_{PSF}({\bar{r}}_{0}-\frac{{z}_{h}}{{z}_{s}}{\bar{r}}_{j}).$$

One can state that the intensity response at the sensor plane is the 2D convolution between the object $O({\bar{r}}_{s})$ and the PSF. The goal here is to reconstruct the object *O* from the camera intensity *I*_*Obj*_. To successfully retrieve the image of the object, let us formulate the intensity response of Eq. (3) as a problem of optical pattern recognition^38–40^. The distribution given by Eq. (3) can be considered as the observed scene in which the patterns of interest *I*_*PSF*_s are distributed according to the shape of the input object. The reconstruction process is done by correlating *I*_*Obj*_ with a reconstructing function calculated based on *I*_*PSF*_, where the goal is to obtain the sharpest delta-like function in each and every position of *I*_*PSF*_ over the entire response *I*_*Obj*_. Next, for a single point object at some ${\bar{r}}_{s},$ the intensity on the camera plane is ${I}_{Obj}({\bar{r}}_{0})={I}_{PSF}({\bar{r}}_{0}-{z}_{h}{\bar{r}}_{s}/{z}_{s}).$ The reconstructing function should be chosen under the constraint that it should be as close as possible to $\delta ({\bar{r}}_{0}-{z}_{h}{\bar{r}}_{s}/{z}_{s}).$ To correlate *I*_*Obj*_ with *I*_*PSF*_ is apparently not the optimal choice, because correlation between two positive functions leads to a high level of background noise, and a correlation peak which is not the sharpest will be achieved. This sub-optimal correlation is equivalent to the use of a matched filter in pattern recognition^39^, and it is demonstrated in the following experiments with a relatively high level of background noise. To choose the optimal reconstructing process we consider the spatial spectral domain, where the Fourier transform of the cross-correlation is a product of the Fourier transforms of *I*_*Obj*_ and the reconstructing function *I*_Re*c*_, as shown in the following:

4 $$\begin{array}{ccc}\mathop{C}\limits^{ \sim } & = & \overline{)\Im }\{{I}_{Obj}\otimes {I}_{{\rm{R}}{\rm{e}}c}\}\\  & = & {\mathop{I}\limits^{ \sim }}_{Obj}{\mathop{{I}^{\star }}\limits^{ \sim }}_{{\rm{R}}{\rm{e}}c}={\mathop{I}\limits^{ \sim }}_{PSF}^{\,}\exp (i2\pi {z}_{h}{\bar{r}}_{s}\,\cdot \,\bar{\nu }/{z}_{s}){\mathop{{I}^{\star }}\limits^{ \sim }}_{{\rm{R}}{\rm{e}}c},\end{array}$$

where $\otimes$ represents 2D correlation, $\bar{\nu }$ is the coordinates of the spatial spectrum, and ${\mathop{I}\limits^{ \sim }}_{Obj}$, ${\mathop{I}\limits^{ \sim }}_{{\rm{R}}{\rm{e}}c}$ and ${\mathop{I}\limits^{ \sim }}_{PSF}$ are the Fourier transforms of ${I}_{Obj}$, ${I}_{{\rm{Re}}c}$ and ${I}_{PSF}$, respectively. Recalling that the purpose is to obtain $C=\delta ({\bar{r}}_{0}-{z}_{h}{\bar{r}}_{s}/{z}_{s})$, the phase of $\mathop{C}\limits^{ \sim }$ should be equal to the linear phase $\exp (i2\pi {z}_{h}{\bar{r}}_{s}\,\cdot \,\bar{\nu }/{z}_{s})$ only, by taking $\arg \{{\mathop{I}\limits^{ \sim }}_{{\rm{R}}{\rm{e}}c}\}=\arg \{{\mathop{I}\limits^{ \sim }}_{PSF}\}$. Hence, the Fourier transform of the cross-correlation becomes,

5 $$\mathop{C}\limits^{ \sim }=|{\mathop{I}\limits^{ \sim }}_{PSF}|\exp (i2\pi {z}_{h}{\bar{r}}_{s}\,\cdot \,\bar{\nu }/{z}_{s})|{\mathop{I}\limits^{ \sim }}_{{\rm{R}}{\rm{e}}c}|.$$

If the equality $|{\mathop{I}\limits^{ \sim }}_{{\rm{R}}{\rm{e}}c}|=|{\mathop{I}\limits^{ \sim }}_{PSF}|$ is chosen, the spatial filter becomes again the matched filter with relatively high background noise and a wide correlation peak. In order to effectively cross-correlate two functions, with relatively low background noise, both magnitudes $|{\mathop{I}\limits^{ \sim }}_{Obj}|$ and $|{\mathop{I}\limits^{ \sim }}_{{\rm{R}}{\rm{e}}c}|$ are raised to a power of *α* and *β*, respectively. Substituting the equality $|{\mathop{I}\limits^{ \sim }}_{{\rm{R}}{\rm{e}}c}|={|{\mathop{I}\limits^{ \sim }}_{PSF}|}^{\beta }$, the Fourier transform of the cross-correlation becomes,

6 $$\mathop{C}\limits^{ \sim }={|{\mathop{I}\limits^{ \sim }}_{PSF}|}^{\alpha }\exp (i2\pi {z}_{h}{\bar{r}}_{s}\,\cdot \,\bar{\nu }/{z}_{s}){|{\mathop{I}\limits^{ \sim }}_{PSF}|}^{\beta }.$$

Note that using the power of α ≠ 1 makes the entire reconstruction process non-linear for a multi-point object^40^. However, we can argue that since *α* does not modify the phase of ${\mathop{I}\limits^{ \sim }}_{Obj}$, but only its magnitude, and since the location of each object point is embedded in the phase distribution, the influence of this non-linearity is mainly an improvement of the SNR of the reconstructed image, as the experimental results show. Recalling that the goal is to obtain $C=\delta ({\bar{r}}_{0}-{z}_{h}{\bar{r}}_{s}/{z}_{s})$, the natural choice for *α* and *β* is the values that satisfy the equation *α* + *β* = 0, a condition that guarantees $|\mathop{C}\limits^{ \sim }|=1$. However, in a practical noisy setup the reconstruction results under this condition are far from being optimal (see the following figures). Therefore, the pair of parameters *α* and *β* should be sought in the range between the inverse filter (*α* or *β* = −1) to the matched filter (*α* = *β* = 1). The search should be based on an optimization of some blind figure-of-merit, since the object in this stage has not been reconstructed yet and, in principle, is unknown to the system user.

For clustered objects on a dark background an appropriate blind figure-of-merit is the entropy^41^. The entropy is maximized when all the energy in the reconstructed image is spread over the entire image matrix, and it is minimized when this same energy is concentrated in the smallest region, i.e., in a single pixel. Therefore, we suggest checking the entropy of the reconstructed image for $-1\le \alpha ,\beta \le 1$ in some chosen step size, and choosing the pair of parameters with the minimum entropy. The entropy corresponding to the energy-normalized distribution function *ϕ* is given as^41^:

7 $$S(\alpha ,\beta )=-\,\sum _{m=1}^{M}\sum _{n=1}^{N}\varphi (m,n)\mathrm{log}(\varphi (m,n)),$$

where $$\varphi (m,n)$$ is,

$$\varphi (m,n)=\frac{{|{\hat{O}}_{mn}|}^{2}}{\sum _{m=1}^{M}\sum _{n=1}^{N}{|{\hat{O}}_{mn}|}^{2}},$$

where M and N are the numbers of rows and columns of the image matrix, and the reconstructed image for a multi-point object is,

8 $$\hat{O}={\overline{)\Im }}^{-1}\{{|{\mathop{I}\limits^{ \sim }}_{Obj}|}^{\alpha }\exp (i[\arg \{{\mathop{I}\limits^{ \sim }}_{Obj}\}-\arg \{{\mathop{I}\limits^{ \sim }}_{PSF}\}]){|{\mathop{I}\limits^{ \sim }}_{PSF}|}^{\beta }\}.$$

Since this search for parameters is done for each object and for each imaging experiment, the system is adaptive for each image and for each noise condition in each experiment. Our experience with the process shows that the two parameters *α* and *β* are different from one object or scene to another. It should be emphasized that the search for the two parameters is done digitally with the same single PSF and with the same single object response. Hence, after the training stage of the system, in which the PSF is stored in the computer, the system captures the object response with only a single camera shot.

## Experiments

**Figure 2:**
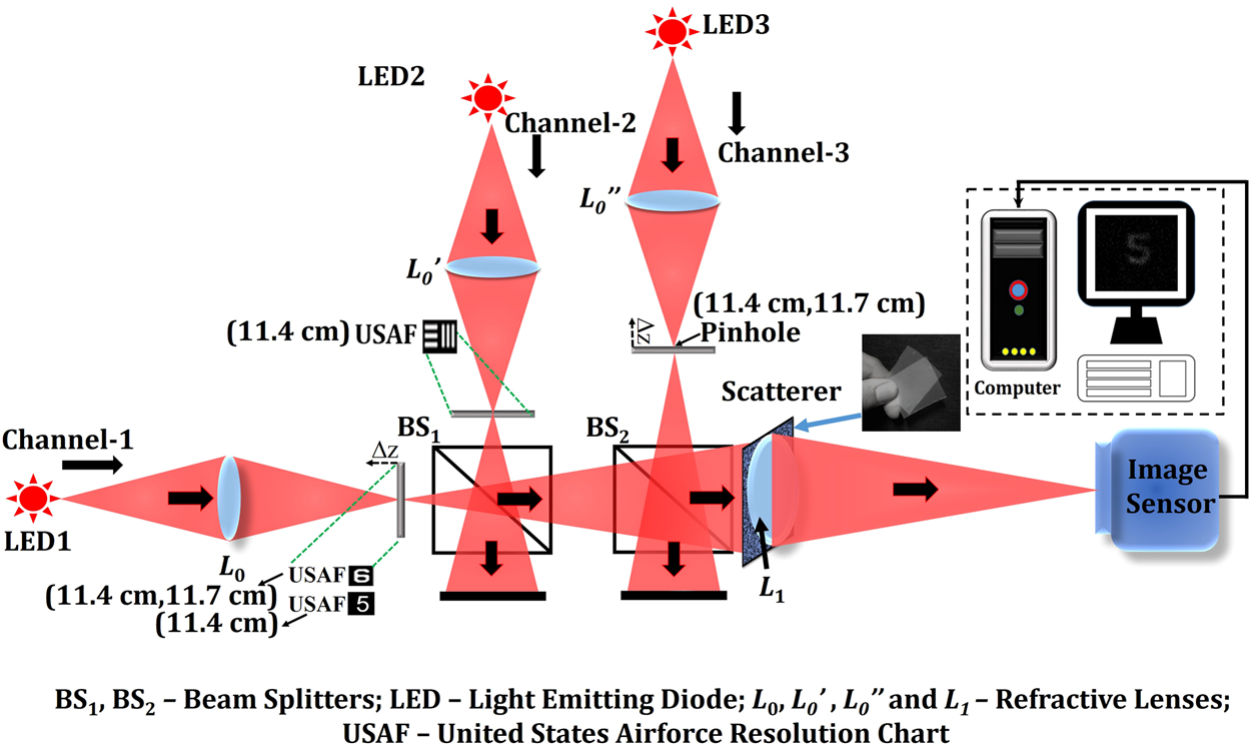
Experimental setup with a scatterer.

The experimental setup is shown in Fig. 2. It consists of three light channels, to facilitate multi-plane imaging, with three LEDs (Thorlabs LED635L, 170 mW, λ = 635 nm, Δλ = 15 nm) serving as incoherent light sources at λ = 635 nm. The channels are adjusted such that the two objects and the point source can be critically illuminated at the same time. Three refractive lenses *L*_0_, *L*_0_′ and *L*_0_′′ were used to illuminate the objects and the pinhole. The experiment was completed in two stages: first, the nonlinear processing was tested using only a single object and the results showed a significant improvement over a linear (*α* = 1) reconstruction process^37^, which encouraged us to proceed to the second part of the experiment, i.e. multi-plane imaging with adaptive tuning. The digit ‘5’ from element 5 of group 2 of the United States Air Force (USAF) resolution chart was considered as the object for the first part of the experiment and a pinhole with an approximate diameter of 100 μm was used as a point object. The object and the pinhole were kept at the same axial location, at a distance of 11.7 cm from the scattering sheet (shown as insert in Fig. 2). A simple polycarbonate sheet (its statistic properties were measured and are described in ref.^37^) was used as a scattering layer, and was placed adjacent to the lens *L*_1_ (focal length 5 cm). The imaging sensor (GigE vision GT Prosilica, 2750 × 2200 pixels, 4.54 μm pixel pitch) was then placed at a distance of 9 cm from the lens *L*_1_, as dictated by the imaging equation. For the multi-plane imaging case, the same pinhole with a different pair of objects was used. Initially, the PSFs were recorded at the two different transverse planes. Next, the object holograms for the multi-plane object were recorded by placing the two different objects in the two channels and separating them by an axial distance of ΔZ = 3 mm. The gratings of element 6 of group 2 in the USAF resolution test chart with 7.13 lp/mm and line spacing of 70.15 μm was considered as object 1, and the numeric digit ‘6’ adjacent to element 6 of group 2 was considered as object 2. All the objects were aligned in the absence of the scatterer, and only once it was ensured that the objects are at the desired axial location, the scatterer was introduced in the setup. An object hologram of a multi-plane object can be reconstructed plane by plane using different pre-recorded PSFs to reconstruct the final image of the original multi-plane object.

The entropies of the reconstructed images were calculated for different values of *α* varying from −1 to +1 in steps of 0.2, and for each individual value of *α*, the filter’s coefficient *β* was tuned between the regime of inverse filter (−1) to matched filter (+1) via phase only filtering (0) with a similar step size of 0.2. The image processing was done off-line after the intensity pattern acquisition stage and where the step size of *α* and *β* can be varied according to the user’s requirement. The non-linear reconstruction for a step size of 0.2 requires only 121 iterations, whereas dropping to a step size of 0.1 the number of iterations increases to 441.

## Experimental Results

**Figure 3:**
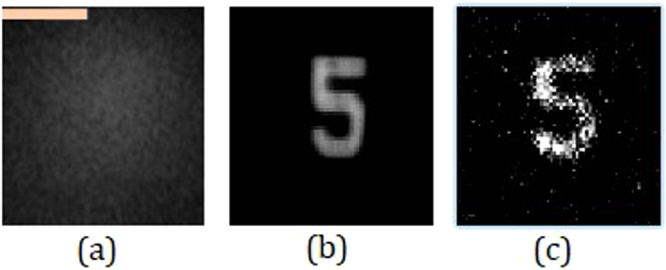
Intensity patterns at the image sensor of (**a**) object with the scatterer in the imaging system and (**b**) object without the scatterer. (**c**) Single shot reconstruction of the object using nonlinear processing. Scale bar: 350 μm.

**Figure 4:**
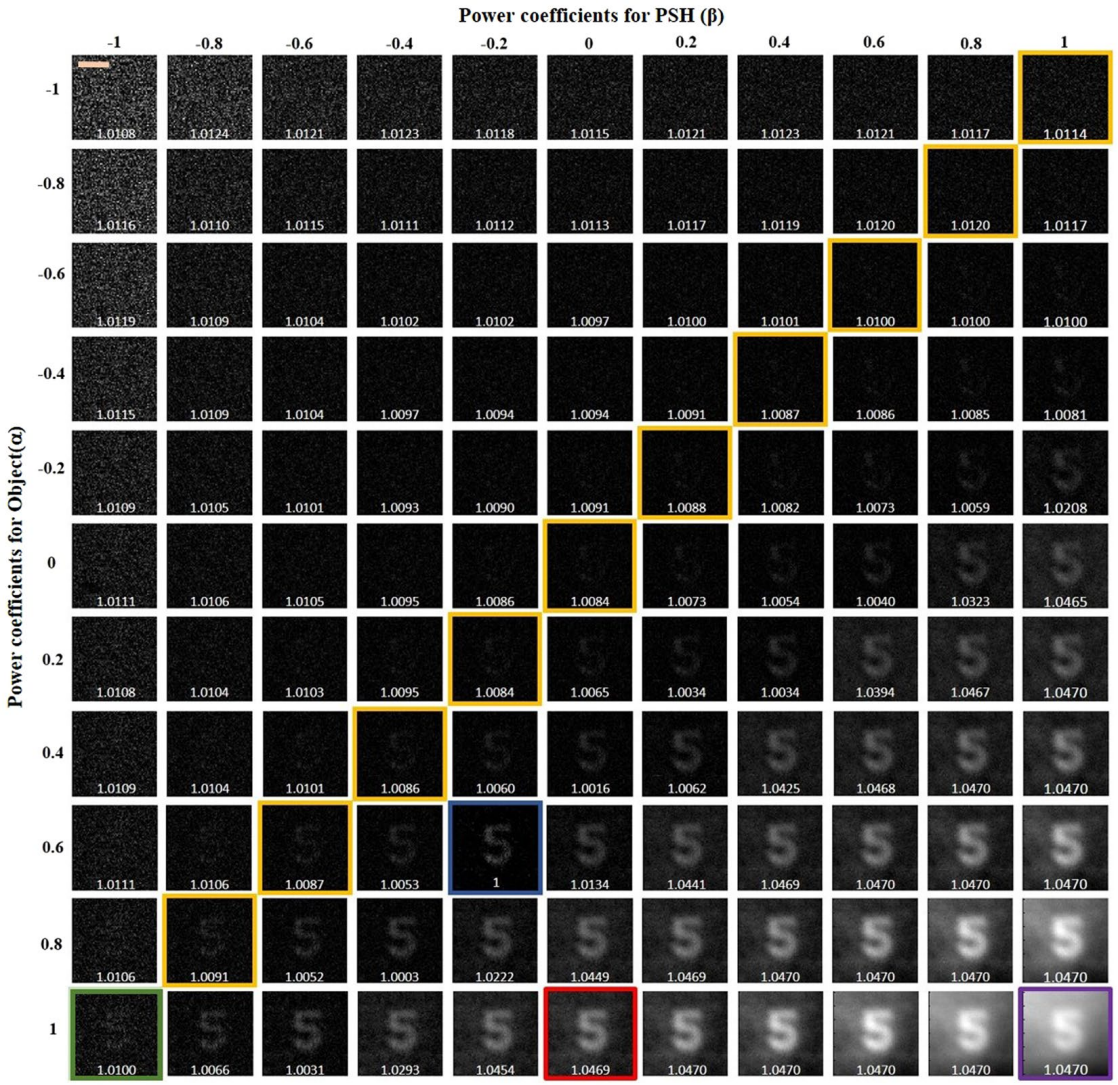
Reconstruction results for different values of *α* and *β*. Scale bar: 350 μm.

The first part of the experiment was carried out by embedding the scatterer between the object and the lens *L*_1_ and recording the intensity profile for the point object ${I}_{PSF}$ and the object ${I}_{Obj}$. The intensity patterns of the object with and without the scatterer are shown in Fig. 3(a,b), respectively. Figure 3(a) is the evidence that the object cannot be seen through the scatterer directly without a digital recovery process. $|{\mathop{I}\limits^{ \sim }}_{Obj}|$ and $|{\mathop{I}\limits^{ \sim }}_{PSF}|$ were raised to the power of *α* and *β*, respectively, and the best reconstruction result with the least normalized entropy value of 1 was obtained for *α* = 0.6 and *β* = −0.2. As explained in the Methodology section, *α* and *β* are the parameters that modify the magnitudes of the object spectrum and the filter, respectively, in order to get the sharpest correlation peak, with minimum sidelobes for every reconstructed image point. *α* and *β* are found in a search procedure, and not analytically, because of the presence of noise (which is different for each experiment) in the spatial spectrum domain. The final reconstructed image with the optimum values of *α* and *β* is shown in Fig. 3(c). The different reconstructed images for different values of *α* and *β*, with their entropy values, are shown in Fig. 4, where the entropy value of each sub-image has been normalized with respect to the minimum entropy value. A few interesting cases are marked with colored outlines. The images that were reconstructed by filtering with a matched filter, a phase-only filter and an inverse filter in a linear (*α* = 1) correlator are designated by purple, red, and green frames, respectively. The entire yellow frames represent the reconstructed images which satisfy the equation *α* + *β* = 0, whereas the optimal case with the least entropy value has been outlined by a blue frame. The same color scheme has been followed throughout the article.

**Figure 5:**
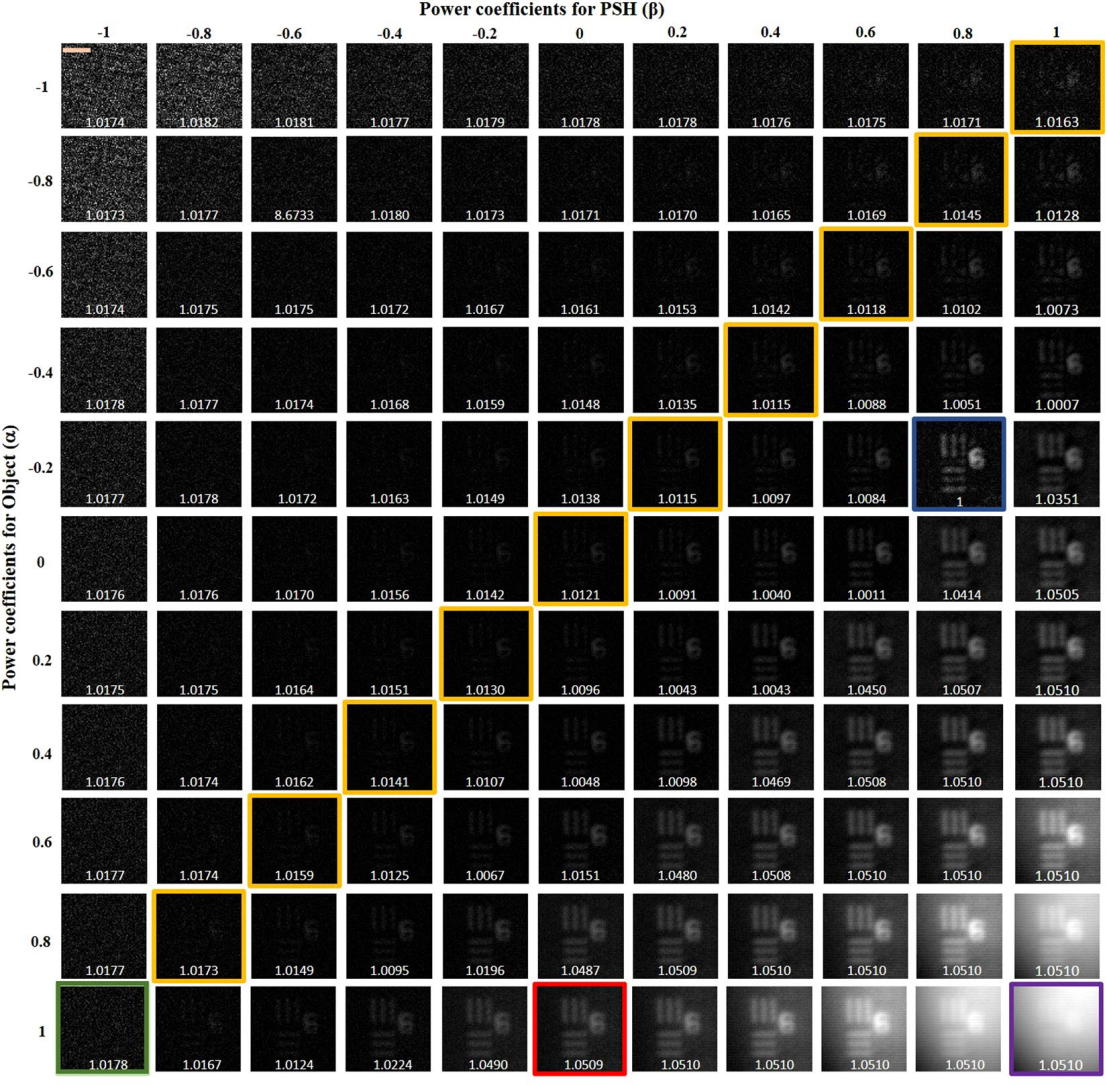
Reconstruction results of the two objects located at the same axial plane for different values of *α* and *β*. Scale bar: 350 μm.

**Figure 6:**
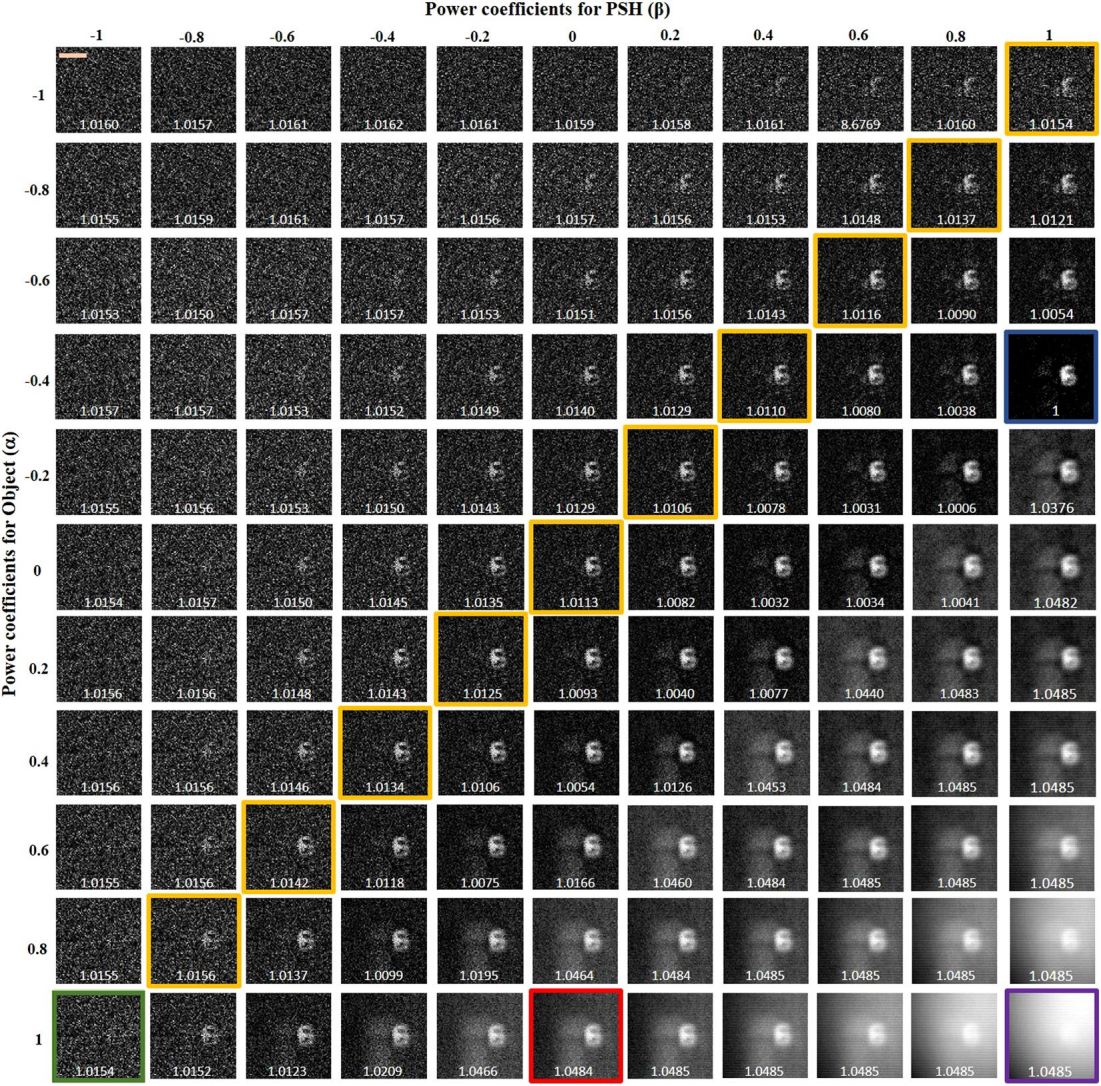
Reconstruction results of the two-plane object using PSF of the axial location of the digit ‘6’, for different values of *α* and *β*. Scale bar: 350 μm.

**Figure 7:**
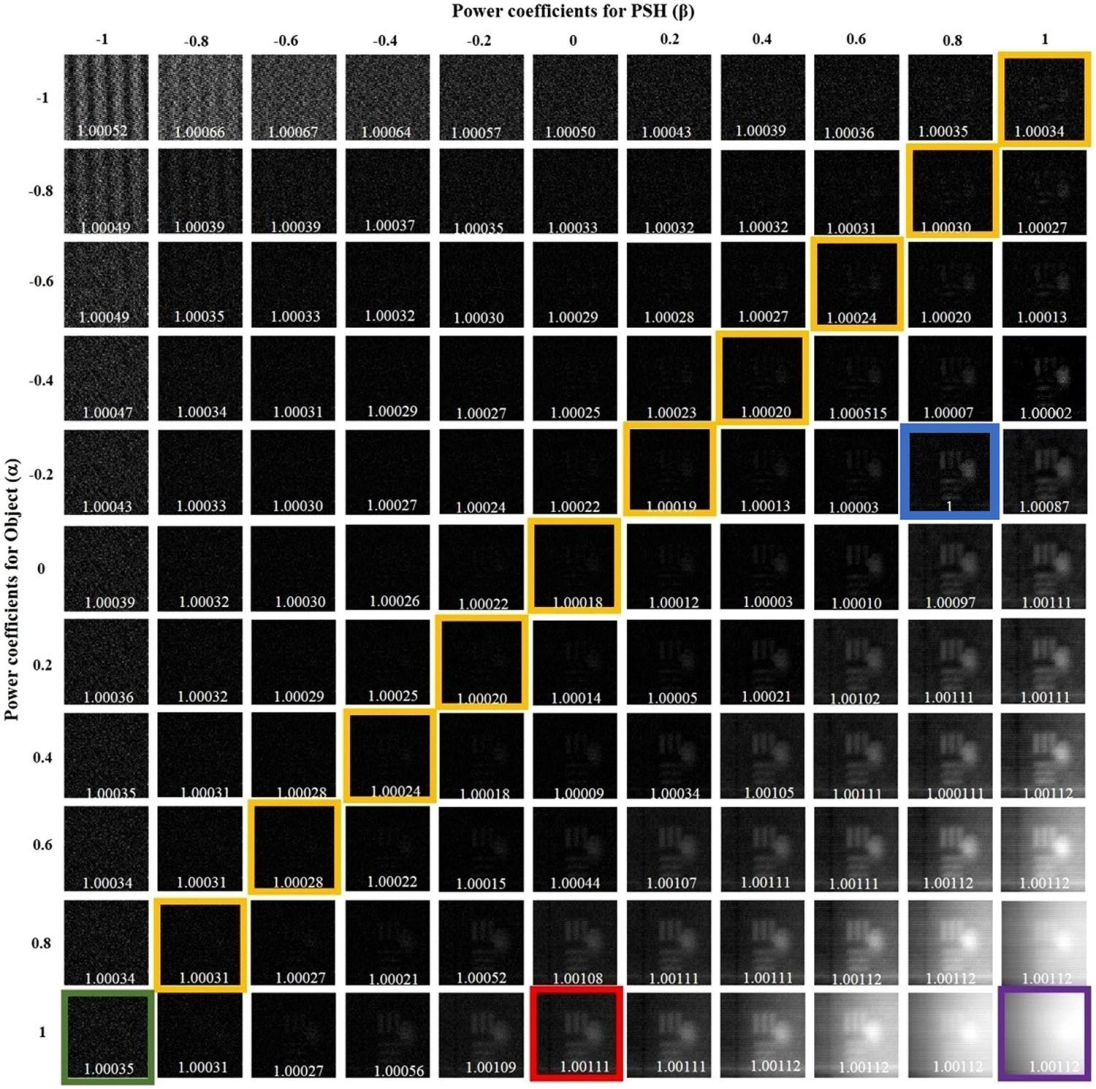
Reconstruction results of the two-plane object using PSF of the axial location of the element 5 of group 2 of the United States Air Force (USAF) resolution chart, for different values of *α* and *β*. Scale bar: 350 μm.

In the next part of the experiment, the multi-plane imaging capability of the system was studied. The two objects mentioned earlier were critically illuminated in two different channels and the pinhole was illuminated in the third channel. Initially, object 1 and object 2 were placed at the same axial location along with the pinhole. All the three objects were kept at a distance of 11.7 cm from the scattering sheet, and the intensities of the object and the pinhole were captured by the image sensor placed at a distance of 9 cm from the lens *L*_1_. The magnitudes of the object and point object were tuned as earlier, and the entropies were calculated. Based on Fig. 5, the best reconstruction result was obtained for *α* = −0.2 and *β* = 0.8. Next, object 1 and object 2 were axially separated by a distance of ΔZ = 3 mm, and the pinhole was kept at the same axial location as that of object 2 (*Z*_2_). The same procedure of tuning *α* and *β* was repeated and the entropies were recorded for different cases. In this case, the best reconstruction result was obtained for *α* = −0.4 and *β* = 1, as shown in Fig. 6. Similarly, when the pinhole and object 1 were in the same axial plane, whereas object 2 was separated by a distance of ΔZ = 3 mm, it was observed that the best reconstructed image was obtained for *α* = −0.2 and *β* = 0.8, as shown in Fig. 7. Note that optimum values of *α* and *β* are different for each state of the 3D scene even when the objects are the same, and hence the non-linear correlation is adaptive in the sense of adapting different optimal *α* and *β* parameters to different observed scenes.

## Summary and Conclusions

In conclusion, we have presented a simple incoherent interferenceless single-shot imaging technique capable of imaging through scattering layers. In this method we have implemented adaptive non-linear processing and demonstrated both single-plane imaging and multi-plane imaging. The scattering layer has to be characterized first by using a guide-star, thus currently making the method effectively invasive imaging through the scattering medium. However, it can be made non-invasive using a fluorescent dye to mark the point object and the object. If such fluorescent markers can be excited from outside the scattering layers, the proposed method might become non-invasive. A pinhole of 100 microns is selected solely to maintain the intensity above the detectable threshold, although a smaller pinhole would provide better image resolution, but might not provide the desirable intensity of light required for the process to work. Thus, while the technique has several advantages, it also has some disadvantages, and additional research is required to overcome them.
